# Precision of the Fully Digital 3D Treatment Plan in Orthognathic Surgery

**DOI:** 10.3390/jcm14144916

**Published:** 2025-07-11

**Authors:** Paula Locmele, Oskars Radzins, Martins Lauskis, Girts Salms, Anda Slaidina, Andris Abeltins

**Affiliations:** 1 Department of the Orthodontics, Institute of Stomatology, Rīga Stradiņš University, Dzirciema Street 20, LV-1007 Riga, Latvia; paula.locmele@rsusi.lv; 2 Baltic Biomaterials Centre of Excellence, Institute of Stomatology, Rīga Stradiņš University, Dzirciema Street 20, LV-1007 Riga, Latvia; oskars.radzins@rsu.lv (O.R.); martins.lauskis@rsusi.lv (M.L.); 3 Department of Oral and Maxillofacial Surgery, Institute of Stomatology, Rīga Stradiņš University, Dzirciema Street 20, LV-1007 Riga, Latvia; girts.salms@rsu.lv; 4 Department of the Prosthodontics, Institute of Stomatology, Rīga Stradiņš University, Dzirciema Street 20, LV-1007 Riga, Latvia; anda.slaidina@rsu.lv

**Keywords:** digital surgical planning, bimaxillary orthognathic surgery, three-dimensional, accuracy

## Abstract

**Background/Objectives:** The aim of this study was to investigate the accuracy of implementing a virtual treatment plan in orthognathic surgery. **Methods:** The study included 30 patients (11 males and 19 females with a mean age of 23.7 years) with a digital surgical plan. All patients underwent bimaxillary orthognathic surgery: LeFort I osteotomy of the maxilla combined with bilateral split sagittal osteotomy (BSSO) of the mandible. Eleven landmarks on the pre-surgical (planned) model and the same landmarks on the post-surgical model were used for comparison and linear difference measurements between the real and predicted outcomes in all three planes—transversal, sagittal, and vertical. **Results:** All median values fell within the 2 mm range in the transversal plane, and the mean displacement was 0.57 mm. In the sagittal and vertical planes, the treatment outcome in the maxilla was more precise than in the mandible. The mean displacement in the sagittal plane was −0.88 mm and that in the vertical plane was 0.44 mm. All deviations were less than 2 mm. **Conclusions:** The data obtained in this study show that the digital surgical plan for orthognathic surgery is clinically reliable in all planes.

## 1. Introduction

Since the beginning of the 20th century, severe dentofacial abnormalities have been corrected by combining different orthodontic and surgical treatments. Orthognathic surgery has developed rapidly in the last few decades, particularly with the development of three-dimensional digital surgical planning, which has become the current standard model for the planning of jaw surgery, including the correction of complex facial asymmetries [1].

The accuracy of orthognathic surgery depends on multiple key factors: the accuracy of dental and skeletal diagnoses, preoperative planning, the transfer of the surgery plan, and the surgical precision, especially in the mandibular region. The digital planning of orthognathic surgery facilitates the surgeon’s work by significantly reducing side effects, as well as improving both the functional and aesthetic results [2,3,4].

Some studies suggest that, in clinical practice, a discrepancy of up to 2 mm between the planned and obtained results of orthognathic surgery is permissible [2,5,6,7,8,9]. Some studies of virtual surgical planning have been published, and most have explored or highlighted the potential benefits of it when compared to conventional methods [2,3,4]. However, research on the accuracy of virtual surgical planning in two-jaw orthognathic surgery appears to be fairly limited [5]. The aim of this study is to investigate the accuracy of implementing a virtual treatment plan in orthognathic surgery in a real-world situation.

## 2. Materials and Methods

In the present retrospective observational study, 30 patients with a digital surgical plan were included (Table 1).

All patients underwent bimaxillary orthognathic surgery in the Riga Stradiņš University Institute of Stomatology between 1 January 2016 and 31 December 2019. All patients had undergone LeFort I osteotomy of the maxilla combined with bilateral split sagittal osteotomy (BSSO) of the mandible. Patients with genioplasty, cleft lip and palate, segmental LeFort I osteotomy, no prior orthodontic treatment, and no cone-beam computed tomography (CBCT) examinations up to 1 month after surgery were excluded. This study was performed in accordance with the Ethical Guidelines provided by the Ethics Committee of Riga Stradiņš University E-9 (2) 2014-02-27.

Measurements of the differences between the planned and real maxillary and mandible positions were performed using linear measurements between the “x” (mediolateral), “y” (anteroposterior), and “z” (vertical) axes. The transversal plane, or the direction of the x axis, is from the left to the right side; the sagittal plane, or the direction of the y axis, is from the posterior to the anterior side; and the direction of the z axis is from the inferior to the superior side.

### 2.1. Preoperative Patient Preparation and Surgical Procedure

Every patient underwent clinical facial analysis, cone-beam computed tomography (CBCT) examination (i-Cat, Next Generation (KaVo Dental GmbH, Biberach, Germany, Imaging Sciences International, Hatfield, PA, USA), 120 kV, 5 mA, 7 s, resolution 0.400, FOV 170 (17 × 23 cm), 891.4 mGy·cm^2^), and intraoral scanning of the dental arches (TRIOS 3, 3Shape intraoral scanner, Copenhagen, Denmark) and had a 3D photo taken (3dMDtrio, Atlanta, GA, USA). Then, 3D-printed dental arch models were created from the intraoral dental scans. These dental models were scanned once again in the preferred final occlusal position. The examination data were used for virtual surgical planning, and individualised 3D virtual surgical plans were developed by two experienced surgeons using the Dolphin 3D Imaging^®^ software (Dolphin Imaging 11.9 Premium and Management Solutions^®^, Chatsworth, CA, USA). A 3D-printed splint for each patient was created according to their virtual plan and was used during surgery.

All patients underwent bimaxillary surgery under general anaesthesia. Surgeries were performed by the same two surgeons from the same maxillofacial surgery centre. All patients underwent BSSO of the mandible followed by a LeFort I osteotomy. Titanium plates and transbuccal screws were used for osteosynthesis. In addition, 2.0 mm KLS Martin (KLS MARTIN^®^, Tuttlingen, Germany) straight profile plates were used for mandibular osteosynthesis, with one on each side. Additionally, transbuccal bicortical screw fixation was performed for the mandibular retromolar region using 10–13 mm positioning screws. Four 2.0 mm L-shaped plates were used for maxillary osteosynthesis. The 3D-printed splint was used to position the mandible in the new position according to the maxilla. Postoperative CBCT was performed for all participants to observe postoperative outcomes.

### 2.2. Data Analysis

After digital orthognathic surgery, two anatomical models were exported from the Dolphin Imaging software—a preoperative model and the planned result model. Both of these models were imported into Autodesk Meshmixer (version 3.5, http://www.meshmixer.com/, accessed on 1 June 2025) to remove soft tissue and hard tissue that was not in the region of interest.

Before and after surgery, CBCT files in DICOM format were imported into Slicer 3D (version 4.10, http://www.slicer.org, accessed on 1 June 2025) [10] and were superimposed using the cranial base as the reference region via a voxel-based algorithm [11].

**Figure 1:**
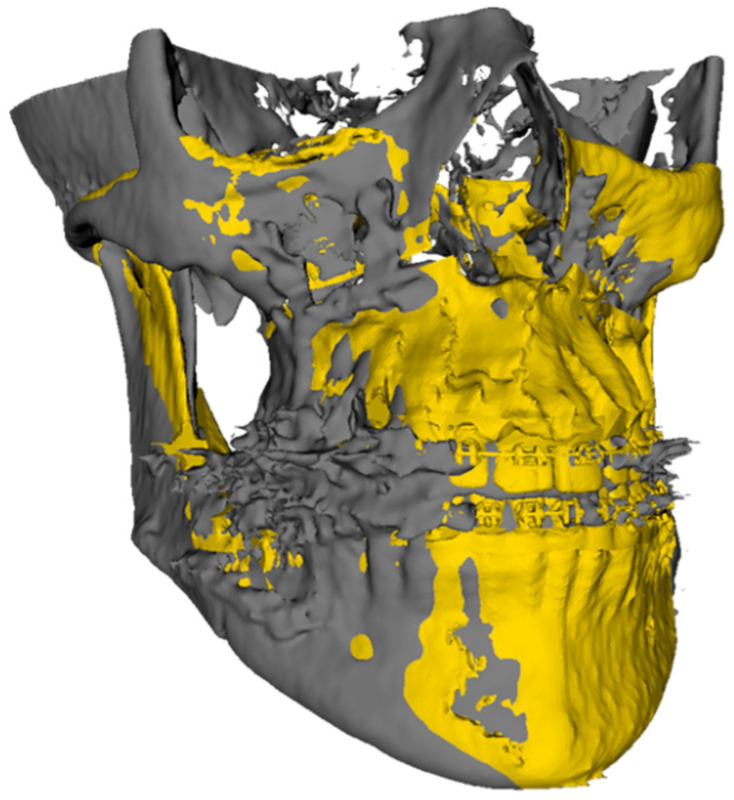
Superimposed pre-surgical planned model (yellow) and post-surgical model (grey).

After superimposition, a model of the hard tissue was created for each time point by applying a thresholding method. The preoperative model from Dolphin Imaging was superimposed on the newly created hard tissue from the preoperative CBCT using region-based superimposition. The regions used were identifiable and surgically unaffected areas, such as the mental foramen, coronoid processes, zygomatic arches, and sigmoid notches, depending on the model quality. The resultant matrix from the region-based superimposition was used to position the planned result model and was therefore superimposed on the actual outcome model (Figure 1).

To improve the accuracy of measurements in the dental region, intraoral scans of the preoperative case were used and positioned in both the superimposed preoperative and postoperative CBCT scans by applying an automatic surface registration method and then performing manual correction in Dolphin Imaging until deemed acceptable.

### 2.3. Measurements

**Figure 2:**
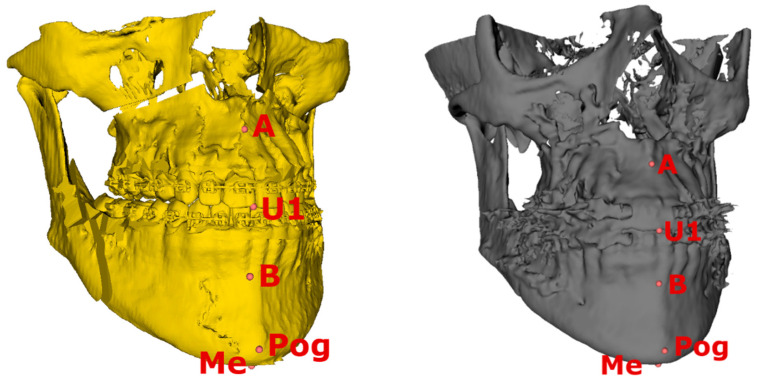
Landmarks (A, B, Pog, Me, U1) from the frontal side on pre-surgical (yellow) and post-surgical (grey) models.

**Figure 3:**
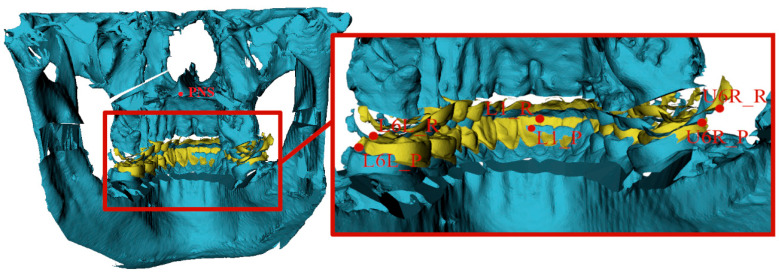
Landmarks (L1, PNS, U6R, U6L, L6R, L6L) from the back side on pre-surgical (yellow) and post-surgical models (blue).

Eleven dental and skeletal landmarks (Figure 2 and Figure 3) were selected—the A point (A), B point (B), pogonion (Pog), menton (Me), upper central incisive (U1), lower central incisive (L1), posterior nasal spine (PNS), upper right first molar (U6R), upper left first molar (U6L), lower right first molar (L6R), and lower left first molar (L6L). All landmarks on the pre-surgical (planned) model and the same 11 landmarks on the post-surgical model were used for comparison and linear difference measurements between the real and predicted outcomes for all three planes (Figure 2 and Figure 3). The Slicer 3D software measured all linear distances automatically. These measurements were chosen to represent the upper (5 landmarks) and lower jaw (6 landmarks). Particular points represented regions with the smallest artefacts from brackets and fixation plates and also were distributed in all 3 planes to allow evaluation in 3 dimensions.

All measurements were assessed by two observers. Ten patients were selected at random for repeated measurements. The assessments were repeated after a two-week interval.

### 2.4. Statistics

A separate group was created for each measured distance projection. Shapiro–Wilk and Lilliefors testing was applied to each group to test for normality, which resulted in a normal distribution not being assumable. The datasets were compared using a Wilcoxon signed-rank test to determine any statistically significant differences between the planned and actual coordinates of the anatomical markings on all axes. An intraclass correlation coefficient (ICC) based on a two-way mixed-effects model was calculated for each point in each dimension to assess the reliability of intraobserver and interobserver measurements, and the results were classified based on recommended criteria [12,13]. All statistics were calculated in R (version 3.6.1, https://www.r-project.org/, accessed on 1 June 2025, R Core Team (2018)).

## 3. Results

Table 2 and Table 3 illustrate the accuracy assessments of the intraobserver repeatability and interobserver agreement tests using the ICC for translational movements at a 95% confidence interval.

The intraobserver repeatability for the x axis was from 0.41 (point PNS) to 0.99 (points U1, L1, L6L). For the y axis and z axis, the lowest values were 0.63 (point PNS) and 0.86 (point A), respectively, and the highest was 0.99 for more than half of the points in both axes. All values were good or excellent, except for the lowest values mentioned previously (Table 2) [12,13].

The ranges of values for the interclass correlation coefficient were 0.73–0.99 for the x axis, 0.84–0.99 for the y axis, and 0.45–0.99 for the z axis. All values were good or excellent, except for points A and B in the z axis (Table 3) [12,13].

### 3.1. Transversal Plane

**Figure 4:**
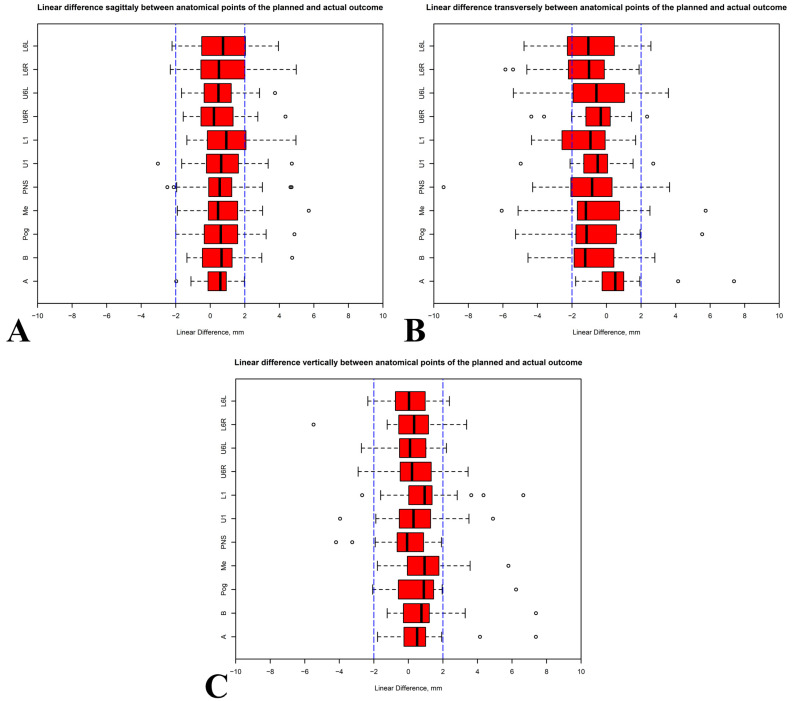
Linear differences between anatomical points of the planned and actual outcomes: (**A**) transversely; (**B**) sagittally; (**C**) vertically.

The direction of the x axis was from the left to the right side. All median values fell within the 2 mm range. The mean displacement of the x axis was 0.57 mm (Table 4). The lowest and highest deviations were for U6R and L1 at 0.21 mm (*p* = 0.096) and 0.93 mm (*p* = 0.002), respectively (Figure 4A; Table 5).

### 3.2. Sagittal Plane

The direction of the y axis was from the posterior to the anterior side. In the sagittal direction on the y axis, the treatment outcome in the maxilla was more precise than in the mandible due to the respective point deviation median values being closer to 0. The mean displacement of the y axis was −0.88 mm. The highest deviation was for B, while the lowest was for U6R, at −1.24 mm (*p* = 0.005) and −0.34 mm (*p* = 0.058), respectively (Figure 4B; Table 5).

### 3.3. Vertical Plane

Similar changes were also observed in the vertical direction on the z axis. The z axis was positioned from the inferior to the superior direction. The displacement of the maxilla was more precise than that of the mandible due to the respective point deviation median values being closer to 0. The mean displacement of the z axis was 0.44 mm. The lowest deviation was −0.08 mm (*p* = 0.919) for PNS and the highest deviations were 0.94 mm (*p* = 0.003) for Me and L1 (Figure 4C; Table 5).

## 4. Discussion

A limited number of articles are available that fully compare the accuracy of digital surgery plans [7,8,9]. In our study, the transverse movement of both jaws was larger than planned; however, the amount of displacement of both jaws on the x axis was similar. The sagittal displacement of both jaws was smaller than planned. Vertically, the displacement of the maxilla was slightly larger than planned, except for point PNS, which exhibited a median difference with a negative value between the planned and real outcomes (−0.08 mm). On the same z axis, the median displacement of the mandible was larger than planned, and the mandibular measurements showed greater inaccuracy than those of the maxilla.

It has been reported that the relocation of an object between CBCT examinations, even if every other parameter remains constant, can lead to a change in intensity levels [14]. Due to the fact that there is some variation in position between the two time points of examination, it is possible that this affects the intensity of the hard tissue, which, in turn, can cause changes in the created model from the segmentation process.

While it has been reported that surface- and voxel-based superimpositions can lead to comparable results, they can only be considered when using the same region of reference [15,16]. In this study, the cranial base was excluded when planning orthognathic surgery; therefore, it could not be used with regard to the planned outcome model. Better results would be expected if the surface superimposition could be avoided by introducing the voxel-based registration of the postoperative DICOM result into the Dolphin Imaging work phase.

Further inaccuracies may stem from the usage of two different segmentation techniques. While both software programs may implement the thresholding method, the smoothing and reduction parameters cannot be controlled in Dolphin Imaging. While Slicer 3D provides more leeway in this regard as it has an adjustable surface smoothing setting, it is not clear how similar the two approaches are. It has been reported that models created using different segmentation software can deviate by up to 0.80 mm when using the same input data [17]. No postoperative intraoral scans were carried out, so no corresponding tooth models could be applied on the operative outcome models. Despite orthodontic treatment being continued after surgery, it is assumed that the movement of the teeth during the time period between the preoperative and postoperative examinations is not significant, as this time period is very small and the patient usually recovers after surgery without active orthodontic tooth movement.

In our study, the average values for all points were less than 2 mm from the planned amount, which means that there was no clinically significant difference between the planned and actual positions [2,5,6,7,8,9]. Discrepancies greater than 4 mm were found for three patients in all three axes. All three of these cases with the largest discrepancies were executed with an older version of the planning software, which could be a reason for the inaccuracy. This previous version of the planning software did not include the so-called “piggyback” function, which enables one to import a digital model of the final occlusion; therefore, it had to be defined manually by placing the jaws in final occlusion virtually under visual guidance. After further analysis of the inaccurate cases, it was discovered that cases with larger asymmetries in the vertical plane also showed larger discrepancies in all planes. In some cases, discrepancies in the vertical plane can be explained by the fact that the required amount of impaction or downgrafting can change during surgery. In these cases, although changes were made only in the vertical plane, it could affect the displacement in all three planes. The mandible showed a larger amount of inaccuracy, which can be explained by the fact that even small deviations from the plan in the vertical plane may cause larger discrepancies, as well as by difficulties with condylar positioning.

One of the most challenging problems in mandibular orthognathic surgery is positioning the condyles in centric relation (CR) during fixation to achieve good accuracy and stability in the postoperative results and optimal temporomandibular joint (TMJ) function [18]. The condylar position changes during general anaesthesia, and it is usually located posteriorly from its position when the patient is awake. This displacement can be explained by the patient’s supine position during surgery and the muscle relaxant drugs that are administered during anaesthesia. Failure to achieve CR during condyle positioning results in immediate relapse after the removal of intermaxillary fixation [19]. Suboptimal condylar positioning can cause the internal derangement of the joint, immediate relapse, an increased risk of late relapse, impaired masticatory function, and condyle sagging [20]. To achieve a stable and precise mandible position, the condyles must be manually placed in a centric-related position, which requires clinical expertise; alternatively, prefabricated positioning guides can be used. All cases in this study involved manual bivectoral condyle positioning performed by an operating surgeon, which is a technique-sensitive procedure. During condylar seating in CR, the surgeon uses both hands and osteosynthesis is carried out by an assistant.

Similar results regarding a mean discrepancy of 0.95 mm in maxillary repositioning between the planned and obtained results for virtual surgical planning have been reported [21]. Another study compared the accuracy of maxillary positioning between conventional model surgery and virtual surgical planning and reported a mean linear difference of 1.44 mm for vertical movements, 0.95 mm for anterior–posterior movements, and 0.90 mm for transverse movements [22]. Other research focused on determining the accuracy of the repositioning of the maxilla. The mean difference in linear measurements was 0.59 mm in the caudal axis, 1.02 mm in the lateral axis, and 1.19 mm in the frontal axis [23]. In another study, all parameters were statistically insignificant, although the mean deviation of point A was greater than 2 mm, which is clinically significant [24]. Further research determined the accuracy of virtual surgical planning in 30 patients, and the overall mean linear difference was 0.81 mm. The same study also showed that the movement of the maxilla (0.71 mm) was more precise than that of the mandible (0.91 mm) in terms of the mean linear difference. It was also found that different methods of transferring virtual plans may affect the precision of the actual outcomes [5].

The use of surgical splints has been the most popular method of transferring surgical plans to actual surgery [2]. In our sample, the surgeons used 3D surgical guides and always began with the mandible. In a published clinical controlled trial in which the accuracy of two navigation techniques—3D surgical guides and a conventional technique using intermaxillary splints—was compared, the highest accuracy was achieved when 3D surgical guides were used [25,26]. However, even with accurate computer-aided design/computer-aided manufacturing (CAD/CAM) surgical splints, it is difficult to obtain precision intraoperatively. There are many rotational and translation movements in all three planes, and the spatial control of these movements during surgery still depends on surgical skill [6].

Despite the promising results obtained in some studies, it has not been possible to compare the methods in the meta-analysis due to the very different approaches used in assessing accuracy and reporting the results, as well as the limited research on determining the accuracy of digital planning [3,6]. The authors of a recent systematic review suggested currently accepted criteria to validate the assessment of the accuracy of digitally planned orthognathic surgery. These three criteria are (1) voxel-based registration on the cranial base; (2) semi-automated or automated evaluation of the outcome; and (3) intra- and interobserver reliability to validate the results [27]. In our study, we used voxel-based registration, but there were also additional steps to combine the coordinate systems. Our distance measurements were semi-automatic because we selected the reference points, but the distance was measured automatically. To check the intra- and interoperator reliability, intraclass and interclass correlations were performed. The intraclass correlation coefficients for all points were obtained, and the ICC was very good for most of the measurements. This is likely due to the intensity changes in the CBCT examination due to the movement of the maxilla, which, in turn, may cause differences when applying segmentation to create the anatomical models. The difference in segmentation methods between the two software programs may also play a part. The lowest values of the interclass correlation coefficient were observed at point A (0.451 on the z axis) and point B (0.624 on the z axis). This could be due to fact that these points are defined two-dimensionally, so they are initially positioned in a sagittal view and then positioned to match the midsagittal plane. However, the surface along the model is not uniform, so moving it transversely may have implications for vertical movement. There were two observers in our study—an orthodontist and a surgeon. There is often minor disagreement between observers when determining the locations of reference points [24]. The points on the CBCT models were more difficult to define because the surfaces were irregular and artefacts from braces and osteosynthesis plates were present.

The data obtained in this study show that the surgical plan for maxillary movement was more predictable and precise when compared to the mandibular plan in the sagittal and vertical planes. The 3D digital planning of orthognathic surgery, if transferred appropriately, can provide an accurate prediction of the surgical outcome, and there is no clinically significant difference between the planned and actual positions. Our digital workflow and methods for superimposition showed the clinically precise transfer of the digital surgical plan to real-life conditions, even in complicated two-jaw surgery cases. In future research, further refinements could be made to the method to enable a more accurate assessment of the outcome of transferring digital surgical plans.

## Figures and Tables

**Table 1:** Summary of the patient cohort included in this study.

Patient Cohort	N = 30
Mean Age	23.7
Gender	
Male	11
Female	19
Class of Occlusion	
Class II	6
Class III	24

**Table 2:** Intraobserver variability and 95% confidence intervals for all landmarks in x, y, and z axes.

	X		Y		Z	
	ICC	95% CI	ICC	95% CI	ICC	95% CI
A	0.80	(0.40; 0.94)	0.98	(0.93; 0.99)	0.86	(0.55; 0.96)
B	0.92	(0.72; 0.98)	0.99	(0.99; 1.00)	0.87	(0.45; 0.96)
Pog	0.97	(0.88; 0.99)	0.99	(0.99; 0.99)	0.93	(0.72; 0.98)
Me	0.95	(0.81; 0.98)	0.93	(0.77; 0.98)	0.90	(0.68; 0.97)
PNS	0.41	(−0.17; 0.80)	0.63	(0.10; 0.89)	0.91	(0.70; 0.97)
U1	0.99	(0.99; 0.99)	0.99	(0.98; 0.99)	0.99	(0.98; 0.99)
U6L	0.98	(0.94; 0.99)	0.99	(0.97; 0.99)	0.99	(0.99; 1.00)
U6R	0.95	(0.81; 0.98)	0.98	(0.93; 0.99)	0.97	(0.89; 0.99)
L1	0.99	(0.98; 0.99)	0.99	(0.97; 0.99)	0.99	(0.97; 0.99)
L6L	0.99	(0.97; 0.99)	0.97	(0.90; 0.99)	0.99	(0.97; 0.99)
L6R	0.98	(0.95; 0.99)	0.99	(0.99; 1.00)	0.99	(0.97; 0.99)

**Table 3:** Interobserver agreement and 95% confidence intervals for all landmarks in x, y, and z axes.

	X		Y		Z	
	ICC	95% CI	ICC	95% CI	ICC	95% CI
A	0.82	(0.65; 0.91)	0.91	(0.82; 0.95)	0.45	(0.10; 0.69)
B	0.79	(0.60; 0.89)	0.96	(0.91; 0.98)	0.62	(0.34; 0.80)
Pog	0.92	(0.85; 0.96)	0.98	(0.97; 0.99)	0.82	(0.67; 0.91)
Me	0.73	(0.51; 0.86)	0.84	(0.70; 0.92)	0.98	(0.97; 0.99)
PNS	0.86	(0.73; 0.93)	0.87	(0.75; 0.94)	0.80	(0.63; 0.90)
U1	0.97	(0.95; 0.98)	0.99	(0.98; 0.99)	0.99	(0.98; 0.99)
U6L	0.99	(0.98; 0.99)	0.98	(0.97; 0.99)	0.98	(0.97; 0.99)
U6R	0.96	(0.91; 0.98)	0.96	(0.93; 0.98)	0.93	(0.85; 0.96)
L1	0.98	(0.97; 0.99)	0.99	(0.99; 0.99)	0.99	(0.99; 0.99)
L6L	0.98	(0.97; 0.99)	0.99	(0.98; 0.99)	0.96	(0.93; 0.98)
L6R	0.97	(0.95; 0.98)	0.98	(0.96; 0.99)	0.94	(0.89; 0.97)

**Table 4:** Linear differences between anatomical points of the planned and actual outcomes.

	X		Y		Z	
	Median, mm	IQR, mm	Median, mm	IQR, mm	Median, mm	IQR, mm
A	0.58	(−0.12; 0.92)	−0.83	(−1.61; 0.03)	0.51	(−0.24; 0.98)
B	0.65	(−0.44; 1.26)	−1.24	(−1.87; 0.37)	0.75	(−0.27; 1.21)
Pog	0.61	(−0.31; 1.55)	−1.16	(−1.75; 0.47)	0.89	(−0.55; 1.45)
Me	0.45	(−0.06; 1.58)	−1.20	(−1.69; 0.70)	0.94	(−0.03; 1.74)
PNS	0.54	(−0.07; 1.23)	−0.84	(−2.02; 0.27)	−0.08	(−0.65; 0.86)
U1	0.63	(−0.19; 1.62)	−0.51	(−1.27; 0.05)	0.30	(−0.52; 1.24)
L1	0.93	(−0.10; 2.03)	−0.93	(−2.37; −0.10)	0.94	(0.02; 1.36)
U6R	0.21	(−0.53; 1.28)	−0.34	(−1.19; 0.20)	0.21	(−0.45; 1.29)
U6L	0.47	(−0.35; 1.19)	−0.59	(−1.83; 0.86)	0.09	(−0.49; 0.97)
L6R	0.50	(−0.53; 1.95)	−1.01	(−2.17; −0.16)	0.33	(−0.53; 1.16)
L6L	0.74	(−0.46; 2.03)	−1.05	(−2.14; 0.29)	0.03	(−0.74; 0.90)

**Table 5:** Comparison of the planned and actual displacement. Statistically significant if adjusted *p* < 0.05 (*).

Point	*p*-Values		
	X	Y	Z
A	0.052	0.015 *	0.067
B	0.083	0.022 *	0.022 *
Pog	0.052	0.082	0.070
Me	0.067	0.057	0.018 *
PNS	0.052	0.048 *	0.159
U1	0.015 *	0.014 *	0.015 *
L1	0.083	0.044 *	0.919
U6R	0.121	0.083	0.128
U6L	0.088	0.210	0.481
L6R	0.072	0.005 *	0.166
L6L	0.044 *	0.028 *	0.785

## Data Availability

All data are available in the main text. The protocols and the anonymised datasets generated and/or analysed during the current study are available from the corresponding author on reasonable request. The patients’ photos and any other identifying information cannot be shared.
